# Evaluating the value of machine learning models for predicting hematoma expansion in acute spontaneous intracerebral hemorrhage based on CT imaging features of hematomas and surrounding oedema

**DOI:** 10.3389/fneur.2025.1567525

**Published:** 2025-06-05

**Authors:** Tianyu Yang, Zhen Zhao, Yan Gu, Shengkai Yang, Yonggang Zhang, Lei Li, Ting Wang, Zhongchang Miao

**Affiliations:** ^1^Department of Radiology, The Affiliated Lianyungang Hospital of Xuzhou Medical University, Lianyungang, China; ^2^Department of Radiology, Affiliated Binhai Hospital, Kangda College of Nanjing Medical University, Yancheng, China; ^3^Jiangsu Key Laboratory of Molecular and Functional Imaging, Department of Radiology, Zhongda Hospital, Medical School, Southeast University, Nanjing, China

**Keywords:** spontaneous intracerebral hemorrhage, hematoma expansion, imaging features, machine learning models, computed tomography scan

## Abstract

**Objective:**

This study evaluates the utility of artificial intelligence (AI) for automated segmentation of intracranial hematomas and surrounding oedema in non-contrast computed tomography (CT) images. Additionally, it aims to extract imaging features for developing machine learning models to predict hematoma expansion in acute spontaneous intracerebral hemorrhage (sICH).

**Methods:**

Data from 183 patients with acute spontaneous hemorrhage, treated at Lianyungang Hospital Affiliated to Xuzhou Medical University between January 2020 and December 2023, were retrospectively analyzed. Patients were divided into training (*n* = 128) and testing (*n* = 55) sets in a 7:3 ratio. CT images were segmented using United Imaging uAI software and both imaging features and clinical characteristics were extracted. Independent risk factors were identified through univariate analysis and least absolute shrinkage and selection operator (LASSO) regression. Machine learning algorithms were applied to construct predictive models for hematoma expansion. Model performance was evaluated using receiver operating characteristic (ROC) curves and the area under the curve (AUC).

**Results:**

Eight feature parameters were extracted from the CT images. The comprehensive model achieved an AUC of 0.9027, with a sensitivity of 0.8235 and specificity of 0.8831. A simplified model utilizing four imaging features yielded an AUC of 0.8897, with a sensitivity of 0.7451 and specificity of 0.9221, slightly underperforming compared to the comprehensive model. Incorporating the subjective ‘swirl sign’, identified as the most significant feature in univariate analysis, into the simplified model enhanced its performance. This optimized model achieved an AUC of 0.9524, with a sensitivity of 0.9412 and specificity of 0.9091, surpassing both the comprehensive and simplified models.

**Conclusion:**

The optimized model, based on CT imaging features of hematomas and surrounding oedema, offers a practical and reliable tool for predicting hematoma expansion in sICH. Its robust performance supports its utility in emergency settings to guide clinical decision-making effectively.

## 1 Introduction

Cerebrovascular diseases are among the leading causes of mortality in urban and rural populations in China, with haemorrhagic stroke accounting for approximately 20–30% of all stroke cases (1). Spontaneous intracerebral hemorrhage (sICH), the most common subtype of haemorrhagic stroke (2), is frequently associated with early hematoma expansion (HE), a critical factor contributing to rapid neurological deterioration and poor clinical outcomes (3). Advances in artificial intelligence (AI) have facilitated the development of predictive models for hematoma expansion using radiomics and deep learning techniques (4, 5). However, the clinical utility of these models has been hindered by non-real-time data processing and non-intuitive outputs. Leveraging advancements in AI-based image segmentation, this study aims to employ automated segmentation of baseline non-contrast computed tomography (CT) scans to extract critical imaging features. These features are then utilized to develop a machine learning model for the rapid and efficient identification of hematoma expansion risks, enhancing decision-making in emergency settings.

## 2 Materials and methods

### 2.1 Study population

**Figure 1:**
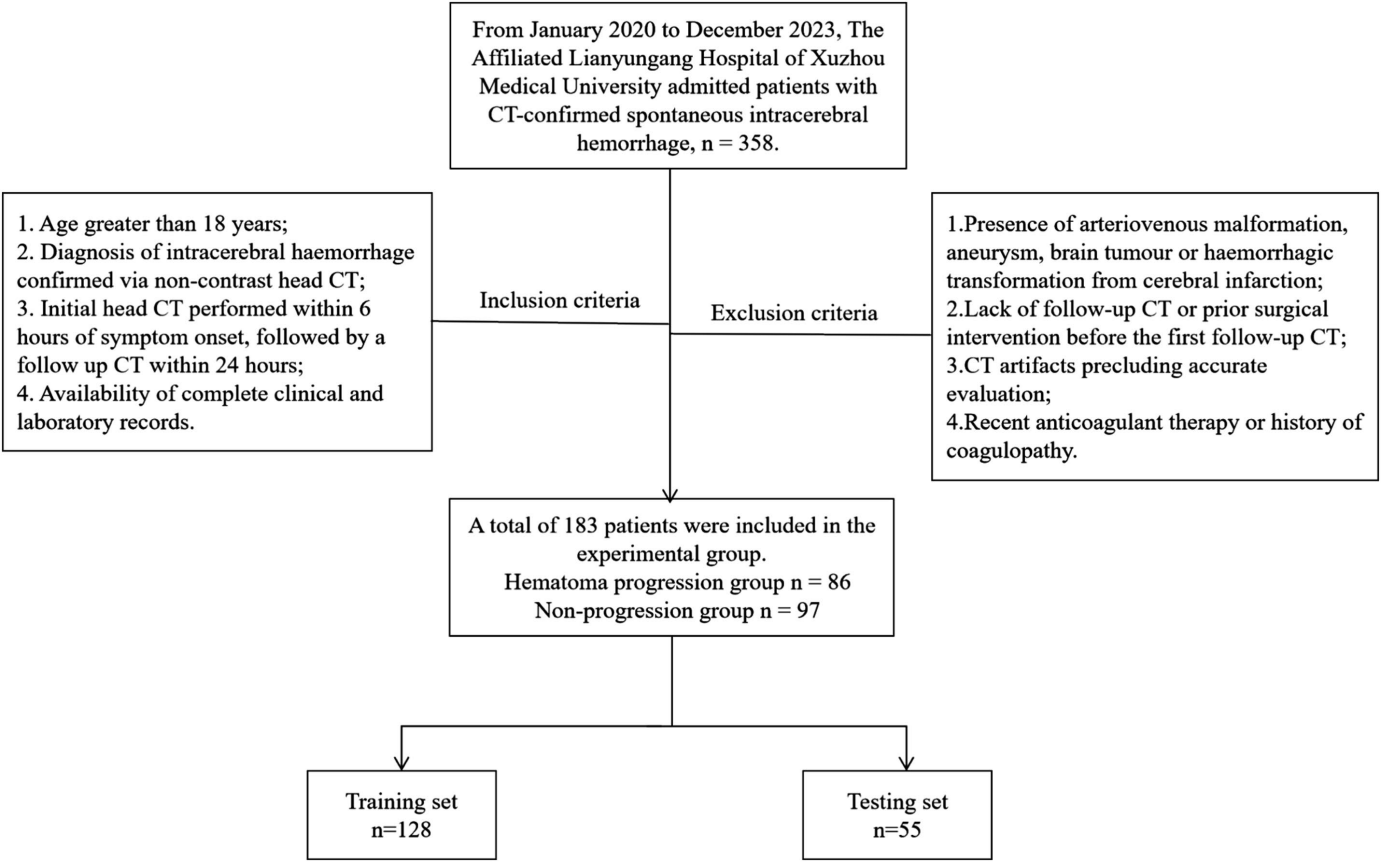
Flow chart for sICH patients.

This retrospective study of patients with sICH admitted to The Affiliated Lianyungang Hospital of Xuzhou Medical University between January 2020 and December 2023. Inclusion criteria were as follows: (1) Age greater than 18 years; (2) Diagnosis of intracerebral hemorrhage confirmed via non-contrast head CT; (3) Initial head CT performed within 6 h of symptom onset, followed by a follow up CT within 24 h; (4) Availability of complete clinical and laboratory records. Exclusion criteria were as follows: (1) Presence of arteriovenous malformation, aneurysm, brain tumor or haemorrhagic transformation from cerebral infarction; (2) Lack of follow-up CT or prior surgical intervention before the first follow-up CT; (3) CT artifacts precluding accurate evaluation; (4) Recent anticoagulant therapy or history of coagulopathy. Patients were classified into expansion and non-expansion groups based on post-recheck CT findings for hematoma expansion, defined as either a ≥ 33% relative increase in baseline hematoma volume or an absolute increase of ≥ 6 mL. Cases were randomly divided into training (*n* = 128) and testing (*n* = 55) sets using the random number generator in the Shukun Research Platform, maintaining a 7:3 ratio. A total of 183 patients with sICH were enrolled in the study. Among them, 86 patients exhibited hematoma expansion, while 97 patients showed no evidence of hematoma expansion (Figure 1).

### 2.2 Imaging protocol

Head imaging was performed using multi-slice spiral CT scanners (Discovery CT750, GE Healthcare, USA). Patients were positioned supine with a head-first orientation. Scans covered the skull base to the vertex, with a tube voltage of 120 kV, tube current of 200 mA, slice thickness of 5 mm, matrix size of 512 × 512 and thin-layer reconstruction at 1.25 mm.

### 2.3 Hematoma imaging segmentation and feature extraction

**Figure 2:**
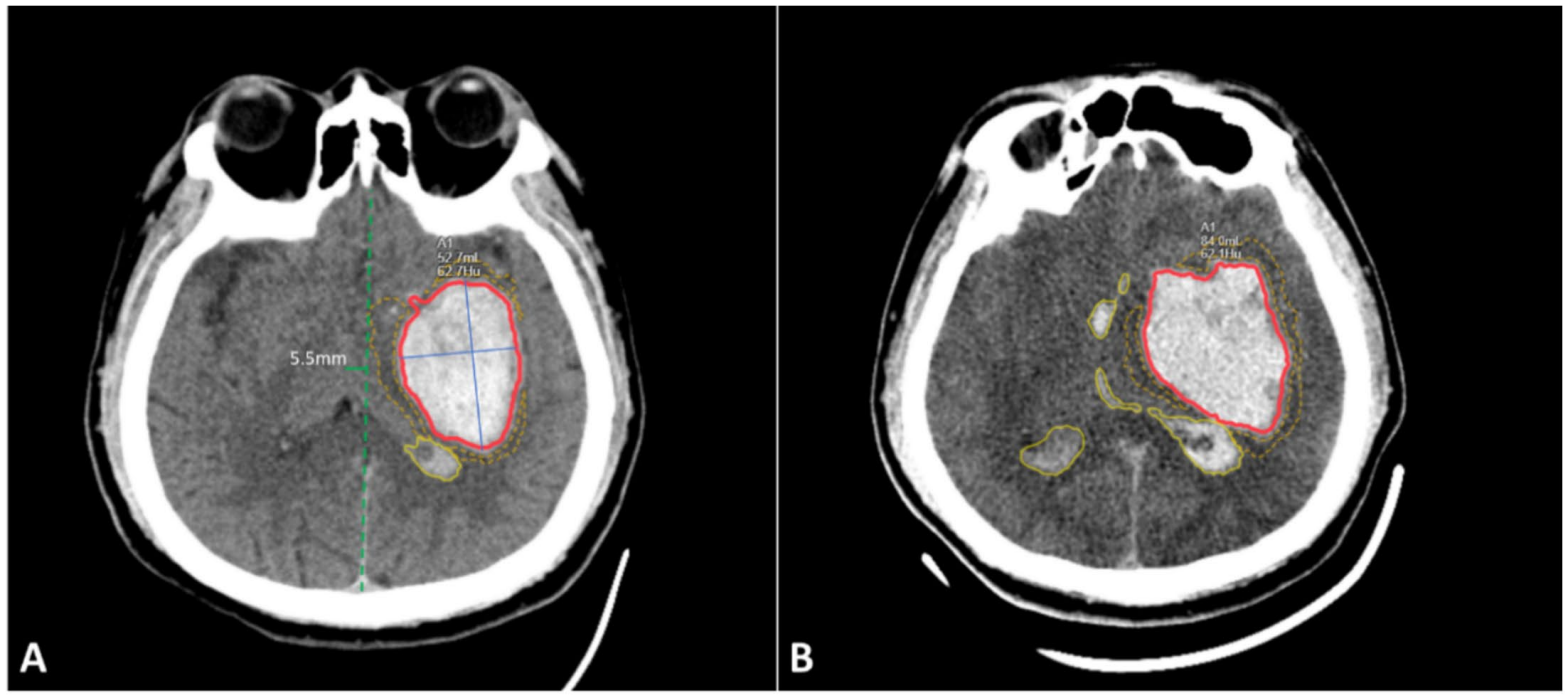
Schematic diagram of imaging feature segmentation. **(A)** Initial non-contrast CT scan images of the brain at 2 h after onset; **(B)** Follow-up CT scan images at 6 h, showing hematoma expansion. Red area: hematoma within the brain parenchyma and its volume; Blue solid line: long and short diameters of the hematoma; Yellow dashed line area: volume of surrounding oedema; Yellow solid line area: hematoma within the ventricles; Green solid line: degree of midline shift. The image in panel **A** shows the presence of the ‘swirl sign’ within the hematoma.

Baseline and follow-up CT images were retrieved from the PACS system in DICOM format and analyzed using United Imaging uAI software. The software automatically segmented hematomas within the brain parenchyma, surrounding oedema, midline structures and other intracranial regions, providing quantitative data, including hemorrhage diameters (short and long), hematoma volume, surrounding oedema volume, ventricular hematoma volume, midline shift and hematoma volume changes (Figure 2). The accuracy of automatic segmentation was reviewed by a senior neuroradiologist with 10 years of experience, who also identified baseline CT imaging features, including irregular shape, hypodensities, swirl sign, black hole sign, blend sign, satellite sign, island sign and fluid level (6–10).

Demographic and clinical data, including age, sex, history of hypertension and diabetes, diastolic and systolic blood pressure, Glasgow Coma Scale (GCS) score and laboratory parameters (white blood cell count, neutrophil count, lymphocyte count, neutrophil-to-lymphocyte ratio [NLR], platelet count, blood glucose, cholesterol, triglycerides, HDL, LDL, prothrombin time, PT activity, international normalized ratio [INR], activated partial thromboplastin time [APTT], thrombin time, fibrinogen and D-dimer), were collected for subsequent analysis.

### 2.4 Statistical analysis

Data analysis was conducted using the Shukun Research Platform. Univariate analyses were performed to evaluate clinical and imaging features. Categorical variables were assessed using the *χ*^2^ test, normally distributed continuous variables using the t-test and non-normally distributed continuous variables using the Mann–Whitney *U* test. Variables with statistically significant differences in univariate analyses (*p* < 0.05) were further analyzed using multivariate logistic regression to identify independent risk factors for hematoma expansion. A *p*-value of < 0.05 was considered statistically significant. Using the selected clinical and imaging features, predictive models were constructed in the training set employing logistic regression, random forest and support vector machine (SVM) algorithms. Model performance was assessed in the testing set by generating receiver operating characteristic (ROC) curves and calculating the area under the curve (AUC).

## 3 Results

A total of 183 patients with spontaneous intracerebral hemorrhage (sICH) were included in the study and randomly divided into a training set (70%) and a testing set (30%). The training set comprised 128 cases, with 51 in the expansion group and 77 in the non-expansion group. A total of 36 features were analyzed, encompassing demographic characteristics (e.g., age, sex), clinical history, laboratory data (e.g., blood pressure, blood glucose, complete blood count, coagulation parameters) and hematoma-related imaging features (e.g., hematoma size, short-to-long diameter ratio, surrounding oedema volume, swirl sign). Detailed data are provided in Table 1.

**Table 1:** Univariate analysis for HE.

Evaluation Metrics	Hematoma expansion		*p* value
	NO (77)	YES (51)	
Demographic			
Age, mean (SD)	57.494 (13.85)	59.157 (13.589)	0.504
Gender (male), *n* (%)	57 (74.0)	33 (64.7)	0.259
Clinical characteristics			
Time to exam, median (IQR)	4.0 (2.0)	4.0 (2.0)	0.814
Diastolic pressure, median (IQR)	94.0 (25.0)	91.0 (18.0)	0.559
Systolic pressure, median (IQR)	158.0 (37.0)	155.0 (32.0)	0.827
GCS, median (IQR)	14.0 (3.0)	13.0 (4.5)	0.153
D-dimer, median (IQR)	146.0 (274.0)	179.0 (258.5)	0.236
FIB(g/L), median (IQR)	2.36 (0.97)	2.5 (1.27)	0.609
INR, mean (SD)	1.058 (0.068)	1.071 (0.084)	0.331
PT, median (IQR)	11.7 (1.0)	11.8 (1.35)	0.629
PT(%), median (IQR)	97.0 (15.0)	95.0 (16.5)	0.378
TT(s), median (IQR)	14.2 (1.8)	14.0 (2.25)	0.806
Platelet, mean (SD)	218.286 (57.936)	202.647 (64.802)	0.156
HDL, median (IQR)	1.18 (0.38)	1.19 (0.36)	0.984
LDL, median (IQR)	2.23 (0.82)	2.48 (0.615)	0.174
LYM, median (IQR)	1.19 (0.75)	1.18 (0.69)	0.423
NEUT, median (IQR)	7.36 (4.01)	7.71 (3.865)	0.733
NLR, median (IQR)	5.991 (5.962)	6.421 (5.339)	0.348
WBC, median (IQR)	9.41 (3.99)	9.17 (3.875)	0.946
Blood glucose, median (IQR)	6.26 (2.9)	6.7 (3.2)	0.336
Cholesterol, median (IQR)	4.14 (1.12)	4.36 (0.955)	0.144
Triglyceride, median (IQR)	1.21 (0.98)	1.21 (0.94)	0.772
Imaging features			
Baseline hematoma volume (ml), median (IQR)	21.6 (20.6)	30.9 (24.6)	0.004
Perihematoma edema volume (ml), median (IQR)	13.6 (12.8)	18.7 (19.35)	0.013
Minor/Major axis, median (IQR)	0.5 (0.125)	0.678 (0.143)	<0.001
Midline shift (mm), median (IQR)	0.0 (4.0)	3.5 (5.5)	0.007
Hypodensities, *n* (%)	29 (37.7)	29 (56.9)	0.033
Swirl sign, *n* (%)	21 (27.3)	27 (52.3)	0.003
Black hole sign, *n* (%)	7 (9.1)	13 (25.5)	0.012
Blend sign, *n* (%)	18 (23.4)	19 (37.2)	0.09
Satellite sign, *n* (%)	1 (1.3)	4 (7.8)	0.16
Island Sign, *n* (%)	4 (5.2)	9 (17.6)	0.022
Broken into ventricle, *n* (%)	15 (19.5)	13 (25.5)	0.421
Hematoma volume in ventricle (ml)	0.0 (0.0)	0.0 (0.25)	0.41
Hemorrhage from other sites, *n* (%)	15 (19.5)	14 (27.5)	0.292
SAH, *n* (%)	0 (0)	2 (3.9)	0.157

GCS, Glasgow Coma Scale; FIB, Fibrinogen; INR, International Normalized Ratio; LYM, Lymphocyte; NLR, Neutrophil-to-Lymphocyte Ratio; SAH, Subarachnoid Hemorrhage.

Univariate analysis revealed no significant differences in demographic or clinical laboratory variables between the expansion and non-expansion groups. However, significant differences were observed in imaging features, including initial hematoma volume, short-to-long diameter ratio, surrounding oedema volume, midline shift distance and the presence of hypodensities, swirl sign, black hole sign and island sign (*p* < 0.05), as shown in Table 1.

**Figure 3:**
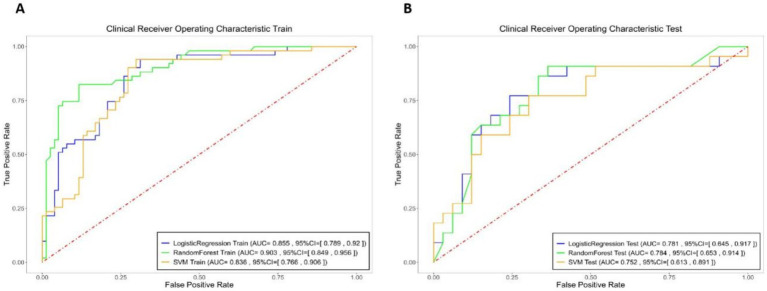
Logistic Regression (blue), Random Forest (green), and SVM (yellow) models specificity, sensitivity, and area under the curve (AUC) of the receiver operating characteristic curve (ROC) for the integrated model in both the training and testing sets.

Using the eight selected imaging features, predictive models for hematoma expansion were constructed with logistic regression, random forest and SVM methods. The performance metrics, including specificity, sensitivity and AUC, for both the training and testing sets, are presented in Figure 3 and Table 2.

**Table 2:** Specificity, sensitivity, and area under the curve (AUC) of the receiver operating characteristic curve (ROC) for the integrated model in both the training and testing sets.

Machine Learning Models	Dataset Type	AUC	SEN	SPE
LogisticRegression	Train	0.8546	0.9412	0.6883
	Test	0.781	0.8636	0.6667
RandomForest	Train	0.9027	0.8235	0.8831
	Test	0.7837	0.7727	0.697
SVM	Train	0.836	0.9412	0.7013
	Test	0.7521	0.8182	0.5152

SVM, Support Vector Machine; AUC, Area Under the Curve; SEN, Sensitivity; SPE, Specificity.

**Figure 4:**
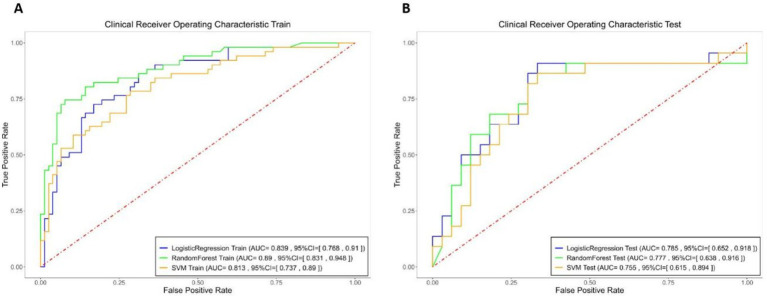
Logistic Regression (blue), Random Forest (green), and SVM (yellow) models specificity, sensitivity, and AUC of the ROC for the objective model in both the training and testing sets.

Among the hematoma characteristics, features such as hypodensities, swirl signs, black hole signs and island signs are subjectively assessed. Therefore, to enhance objectivity, we constructed the hematoma expansion model using four purely objective imaging indicators. The specificity, sensitivity and AUC of these models for the training and testing sets are summarized in Figure 4 and Table 3.

**Table 3:** Specificity, sensitivity, and AUC of the ROC for the objective model in both the training and testing sets.

Machine Learning Models	Dataset Type	AUC	SEN	SPE
LogisticRegression	Train	0.8388	0.7255	0.8312
	Test	0.7851	0.7273	0.697
RandomForest	Train	0.8897	0.7451	0.9221
	Test	0.7769	0.7273	0.697
SVM	Train	0.8133	0.7843	0.7143
	Test	0.7548	0.8182	0.6667

SVM, Support Vector Machine; AUC, Area Under the Curve; SEN, Sensitivity; SPE, Specificity.

Additionally, based on univariate analysis, the swirl sign—identified as having the smallest *p*-value—was combined with the four objective imaging indicators to develop an enhanced hematoma expansion model. The specificity, sensitivity and AUC for this model in both the training and testing sets are also detailed in Table 4.

**Table 4:** Specificity, sensitivity, and AUC of the ROC for the optimized model in both the training and testing sets.

Machine Learning Models	Dataset Type	AUC	SEN	SPE
LogisticRegression	Train	0.8531	0.8824	0.7143
	Test	0.7576	0.8636	0.5455
RandomForest	Train	0.9524	0.9412	0.9091
	Test	0.7507	0.7727	0.6667
SVM	Train	0.8139	0.6863	0.8052
	Test	0.7163	0.6818	0.6364

SVM, Support Vector Machine; AUC, Area Under the Curve; SEN, Sensitivity; SPE, Specificity.

**Figure 5:**
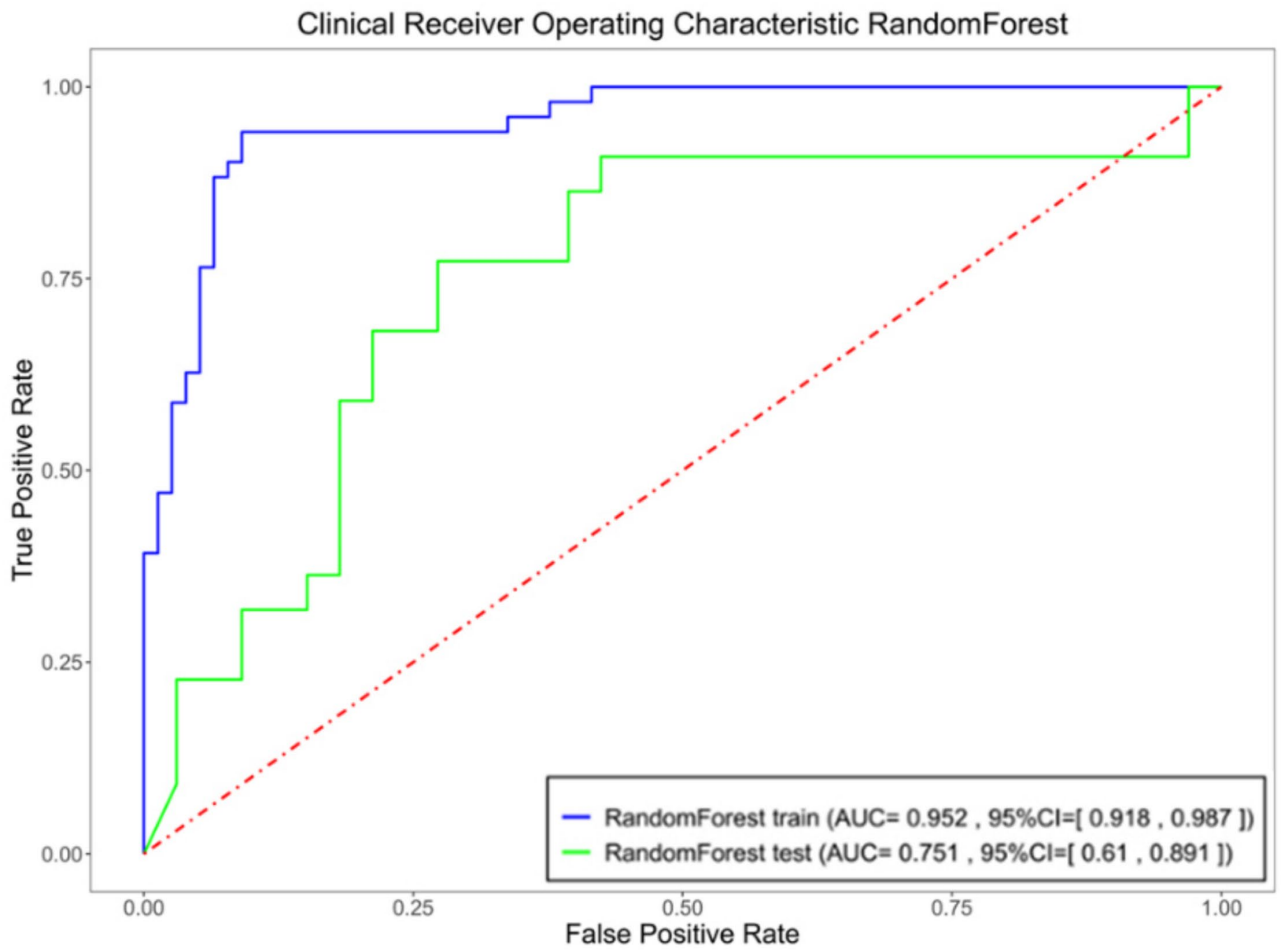
Specificity, sensitivity, and AUC of the ROC for the optimized model in both the training and testing sets.

The random forest algorithm, which demonstrated superior performance based on AUC results, was selected for further analysis. The ROC curves for the random forest model in the training and testing sets are depicted in Figure 5.

**Figure 6:**
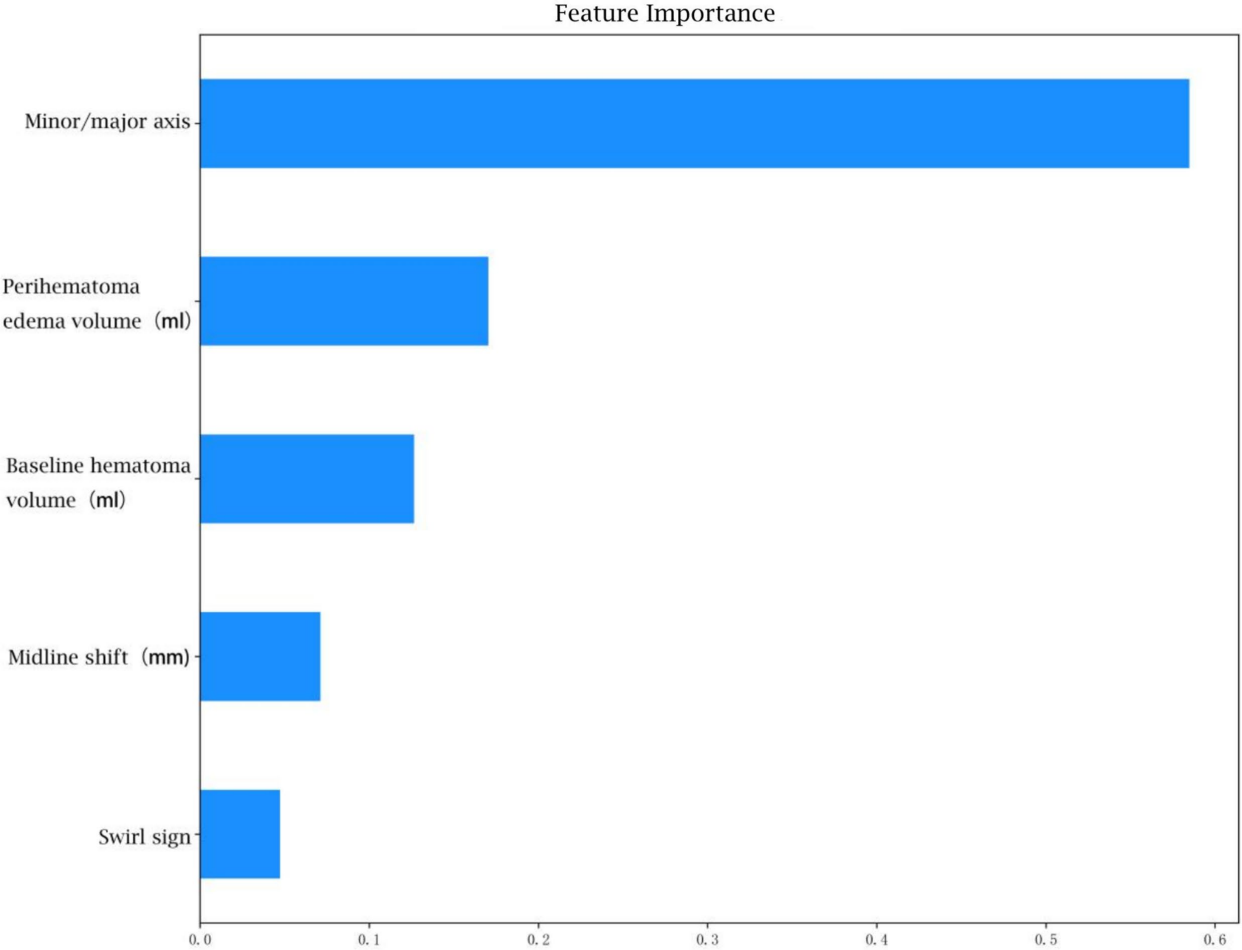
Weighting factors for each clinical characteristic in the optimized model.

The feature weight coefficients are displayed in Figure 6 and Table 5.

**Table 5:** Weighting factors for each clinical characteristic in the optimized model.

Feature_name	Importance
Baseline hematoma volume (ml)	0.126564
Midline shift (mm)	0.071180
Perihematoma edema volume (ml)	0.170349
Minor/major axis	0.584698
Swirl sign	0.047209

## 4 Discussion

Spontaneous intracerebral hemorrhage (sICH) is a severe condition characterized by rapid onset and expansion, resulting in significant disability and mortality rates that profoundly affect patients’ quality of life (11). Hemorrhage expansion complicates treatment decisions, exacerbates neurological deficits and often leads to unfavorable outcomes, including death (12). Consequently, the early and accurate identification of hematoma expansion is critical for guiding clinical interventions, reducing neurological damage and improving patient prognosis.

CT remains the imaging modality of choice for sICH due to its rapid acquisition speed and high sensitivity in detecting lesions (13). Previous studies have identified several non-contrast CT imaging features, such as the island sign, black hole sign, blend sign and irregular sign, as independent predictors of hematoma expansion (8, 14). However, these features rely heavily on subjective interpretation, requiring significant expertise and experience, which limits their consistency and generalizability. Therefore, developing an objective, automated and accurate prediction model is imperative for advancing ICH management.

In this study, we utilized an AI-based image segmentation model to automate the analysis of non-contrast CT images, enabling the rapid and accurate quantification of hematoma volume, diameters and surrounding oedema. This automated approach demonstrated high reliability and effectively minimized human errors associated with slice selection and manual measurement (15).

To construct a robust prediction model, we integrated readily available clinical data, including patient history and laboratory results, with imaging features derived from CT scans. Univariate analysis revealed no statistically significant differences in demographic or laboratory variables, suggesting that these factors have limited predictive value for hematoma expansion.

Through LASSO regression, we identified eight key imaging features—initial hematoma volume, short-to-long axis ratio of the hematoma, surrounding oedema volume, midline shift distance, hypodensities, swirl sign, black hole sign and island sign—for inclusion in a ‘comprehensive model’. This model achieved an AUC of 0.9027, with sensitivity and specificity values of 0.8235 and 0.8831, respectively, indicating strong predictive performance. However, the inclusion of subjective features such as hypodensities, swirl sign, black hole sign and island sign introduced variability dependent on the diagnostician’s expertise. To enhance objectivity, we developed an ‘objective model’ by excluding subjective features and retaining only quantitative indicators: initial hematoma volume, short-to-long axis ratio, surrounding oedema volume and midline shift distance. This model achieved an AUC of 0.8897, sensitivity of 0.7451 and specificity of 0.9221, demonstrating slightly reduced predictive performance compared to the comprehensive model. Subsequently, we constructed an ‘optimized model’ by reintroducing the swirl sign—a feature with the smallest *p*-value in univariate analysis—alongside the four objective indicators. The optimized model achieved an AUC of 0.9524, with sensitivity and specificity values of 0.9412 and 0.9091, respectively, demonstrating superior predictive accuracy compared to the other models. On the test set, this model achieved an AUC of 0.7507, sensitivity of 0.7727 and specificity of 0.6667.

In the analysis of feature weights within the ‘optimized model’ for predicting hemorrhage expansion, the short-to-long axis ratio of the hematoma emerged as the most influential imaging feature, contributing a weight of 0.58. A larger ratio, approaching 1, correlates with a higher likelihood of expansion, reflecting a more rounded or irregular hematoma shape. This morphology suggests outward expansion and increased tension. Conversely, a smaller ratio, indicative of an elongated hematoma, implies expansion along interstitial spaces in brain tissue, associated with lower tension (16). Thus, the short-to-long axis ratio serves as a critical indicator of hematoma morphology and expansion potential. Previous studies have established a strong relationship between initial hematoma volume and ICH expansion and prognosis, with larger initial volumes associated with higher early hemorrhage rates (12). The timing of imaging relative to symptom onset further reinforces this relationship, as larger hematomas typically reflect greater instability (17). Surrounding oedema volume, another significant feature, is closely linked to hematoma size (18). Some studies also highlight its association with CTA indicators of hemorrhage expansion, suggesting that surrounding oedema volume may indirectly reflect the likelihood of hematoma expansion (19). Midline shift distance, representing the hematoma’s mass effect, correlates with hematoma volume, surrounding oedema and the rate of expansion. Consequently, it serves as an important marker of hemorrhage expansion (20).

Subjective imaging features such as hypodensities, swirl signs, black hole signs and island signs have been widely recognized as independent predictors of hemorrhage expansion. However, their interpretation is subject to significant variability, heavily reliant on clinician expertise and experience. Studies have reported substantial inter-rater variability for these features, though the swirl sign demonstrates relatively higher consistency (8). In our univariate analysis, the swirl sign showed the most statistically significant association with hemorrhage expansion. While the comprehensive model, which incorporates all four subjective features, exhibited strong predictive efficacy, the optimized model—including only the swirl sign—achieved improved performance. This enhancement is likely due to the swirl sign’s superior diagnostic consistency.

Compared to previous analogous studies (21, 22), our model demonstrates superior accuracy and incorporates AI-driven automated segmentation, significantly enhancing its clinical applicability.

Despite these promising findings, this study has limitations. It is a single-center retrospective analysis with a limited sample size, introducing potential selection bias. Additionally, the timing of CT scans relative to symptom onset was uncertain in some emergency ICH cases, potentially affecting the accuracy of initial imaging features. Future research should employ prospective, multicenter studies with larger sample sizes to validate the reliability and generalizability of the proposed model.

## 5 Conclusion

This study confirms the effectiveness of an objective feature model derived from non-contrast CT images in predicting hematoma expansion in spontaneous sICH. The inclusion of the swirl sign, a feature with high diagnostic consistency, further enhances the model’s predictive performance. The objective indicators-initial hematoma volume, short-to-long axis ratio of the hematoma, volume of surrounding oedema and midline shift distance-can be rapidly and accurately quantified using AI-driven image segmentation models, ensuring intuitive and reliable results.

## Data Availability

The raw data supporting the conclusions of this article will be made available by the authors, without undue reservation.
